# Quality Assessment of Services in Primary Healthcare in Iran: A Systematic Review and Meta-analysis

**DOI:** 10.4314/ejhs.v32i2.26

**Published:** 2022-03

**Authors:** Hojjat Rahmani, Raheleh Maleki, Mahboubeh Khaton Ghanbari, Masoud Behzadifar

**Affiliations:** 1 Department of Health Management and Economics, School of Public Health, Tehran University of Medical Sciences, Tehran, Iran; 2 PHD candidate of health management, Department of Health Management and Economics, School of Public Health, Tehran University of Medical Sciences, Tehran, Iran; 3 Social Determinants of Health Research Center, Lorestan University of Medical Sciences, Khorramabad, Iran

**Keywords:** Primary health care, Quality assessment, SERVQUAL instrument, Systematic review, Meta-analysis, Iran

## Abstract

**Background:**

Primary healthcare (PHC) plays an important role in achieving universal health coverage (UHC). The SERVQUAL instrument is the tool for evaluating the quality of services in the health sector. The main purpose of this study is to evaluate the quality of services provided in PHC in Iran using the SERVQUAL instrument.

**Materials and Method:**

We searched eight databases from January 2000 to September 2021. We analyzed the mean of various SERVQUAL instrument items using the DerSimonian-Laird approach via a random model with 95% confidence interval. Also, we used I2 to evaluate the heterogeneity of the studies.

**Results:**

Finally, 17 studies were chosen for analysis in the present study. There were 8,767 study participants, out of which 8,237 were female and 530 were male. The mean dimensions of perception were as follows: Tangibles = 3.71, reliability = 4, responsiveness = 3.79, assurance = 3.83, and empathy = 3.86. For the expectation, the mean dimension were: Tangibles = 4.46, reliability = 4.46, responsiveness = 4.36, assurance = 4.36, and empathy = 4.36 respectively. The total gap quality between perception and expectation was -0.53.

**Conclusion:**

All dimensions of quality based on SERVQUAL were negative, and the quality of service in PHC is not satisfactory. Therefore, policymakers must adopt serious and effective programs to improve services in this area. We also recommend that quality management of services in PHC in Iran should move toward comprehensive optimization in all areas, and quality in this area should be a priority.

## Introduction

Primary healthcare (PHC) services are one of the most important components of health systems in any country that play an important role in achieving universal health coverage (UHC) (1). PHC is an approach that affects the whole community, and its goal is to achieve the highest level of health, equitable distribution of services, and respond to the needs of all members of the community that would ultimately improve the health (2). By increasing costs and out-of-pocket payments for health-related services, increasing PHC interventions can be an important policy to reduce costs and improve the health index in lowand middle-income countries (3). Thus, health policymakers can gain significantly in the health sector by investing more in PHC (4, 5).

Promotion, regular evaluation, and continuity of the quality of services provided in various health sectors are among the concerns of policymakers (6, 7), who are trying to ensure the high quality of health services by implementing various programs (8). The health activities, due to their nature and impact on individuals' life, should be of high quality, and evaluate continuously so that their quality does not decline (9, 10). Health service providers, on the other hand, are competing for greater customer satisfaction (11). Due to the importance of the role of PHC in promoting health, the high quality of services provided in these centers can further encourage the community to use the services and effective policies (5, 12).

Iran, through investing in PHC, has had valuable successes in the health sector in recent years. PHC services began in 1980 in Iran, from villages to various cities (13, 14). Various services such as vaccination, assessment of the condition of pregnant women during pregnancy, health education, water and food hygiene, workers' health, and screening for various diseases are provided in PHC (15). High vaccination coverage, elimination of several infectious diseases, increasing life expectancy, and reducing child and maternal mortality have been some of the valuable achievements gained via PHC in Iran (16, 17).

In all provinces in Iran, medical universities are responsible for providing the medical services for the health system, as well as improving the quality of services in order to get people involved in decision-making, planning, and using their cooperation in providing healthcare (18). Currently, there are more than 24,000 health units in Iran that are supervised by medical universities across the country. Easy access to PHC services by everyone was the most important goal of the development of this large and successful network in the country. With the development of PHC and the provision of services, significant achievements have been gained for the Iranian health system (19); for example: Life expectancy increased from 50.9 to 75.7 between 1970 and 2015; maternal mortality ratio has decreased from 255 to 25 per 100,000 during these years; infant mortality rate has also decreased from 125 to 13.8 per 100,000 births; vaccination coverage in Iran has reached over 95%; mortality rates from infectious diseases have dropped dramatically, and HIV testing has progressed as well. In addition, with public education provided by PHC staff, the public awareness of diseases has increased (20).

Various studies have been conducted to evaluate the quality of services provided in PHC in Iran. In this regard, we believe that findings of the present study can help reveal the status of service quality in PHC, and give policymakers a more comprehensive view of the current situation. It can also be a roadmap for implementing new interventions in order to improve the quality of PHC services in Iran. Thus, we set out the present study to evaluate the quality of services provided in PHC in Iran, using SERVQUAL instrument.

SERVQUAL instrument was widely used to assess the quality of services in the health sector in several countries. Parasuraman et al. first introduced SERVQUAL based on the gap theory of service quality (21). SERVQUAL assesses the quality of services provided in two areas of expectations and perceptions of service recipients, as well as in the following aspects: Tangibles, reliability, responsiveness, assurance, and empathy. Total service quality and quality gap can also be calculated via SERVQUAL.

## Materials and Method

In this systematic review, we followed the “Preferred Reporting Items for Systematic Reviews and Meta-Analyses” (PRISMA) guidelines (22). (Appendix 1).

**Search strategy**: We searched the Medline via PubMed, Embase, Web of Sciences, The Cochran library, and Scopus, as well as the Iranian MagIran, Scientific Information Database (SID) and Barakatkns databases from January 2000 to September 2021. The two authors of this study performed this step independently using related keywords. The keywords used for the search were (SERVQUAL model, SERVQUAL tool, SERVQUAL, SERVQUAL evaluation model, SERVQUAL instrument) AND (Primary health care, PHC, health services, service quality, health quality) AND (Iran).

**Inclusion criteria**: We included articles that met the following criteria 1) The articles were conducted in PHC centers in Iran, and their SERVQUAL tool was used to evaluate the quality of their services, 2) Articles published in peer-reviewed journals, 3) Articles published in English and Persian, 4) Articles whose working methods were clearly stated and their findings were available, 5) Articles that reported all SERVQUAL instrument items (expectations and perceptions of service recipients), 6) Articles that were cross-sectional designed and conducted, 7) Articles that reported mean and standard deviation of SERVQUAL instrument items and 8) Articles whose full text was available.

**Exclusion criteria**: We excluded articles that met the following criteria 1) Studies conducted in hospitals, 2) Published studies whose findings overlapped, 3) Studies whose findings were unclear or not fully reported, 4) Studies published as a letter to the editor brief reports, qualitative studies, and reviews, 5) Abstracts of papers presented at conferences and seminars.

All authors agreed upon all the inclusion and exclusion criteria.

**Assessment of study quality**: The quality of the methodology of the selected studies was assessed by the critical appraisal skills program (CASP) checklist (23). This checklist has 10 questions with three items: Yes, No, and Unclear. Two authors of the present study evaluated the quality of the methodology of the selected studies independently. Disagreements between them were then resolved by the third author through discussion.

**Data extraction**: In this step, we first extracted the name of the first author, year of publication, geographical location of the study, number of participants, gender of participants, mean age of participants, mean and standard deviation of expectations, and perceptions and quality gap. Preliminary data of the studies selected by the two authors were extracted independently, and their differences were resolved through discussion through a third party.

**Statistical analysis**: The mean of different SERVQUAL instrument items was analyzed using the DerSimonian-Laird approach via a random model with 95% confidence interval (CI) (24). Also, we used I2 to evaluate the heterogeneity of the studies. Meanwhile, we used Egger test to assess the publication bias (25). Sensitivity analysis was performed for all items to ensure consistency of results. All analyses were conducted using STATA Version 12 (STATA Corp, College Station, TX, USA). Also, in all the statistical analyses, figures with a p-value less than 0.05 were considered statistically significant.

## Results

**Figure 1:**
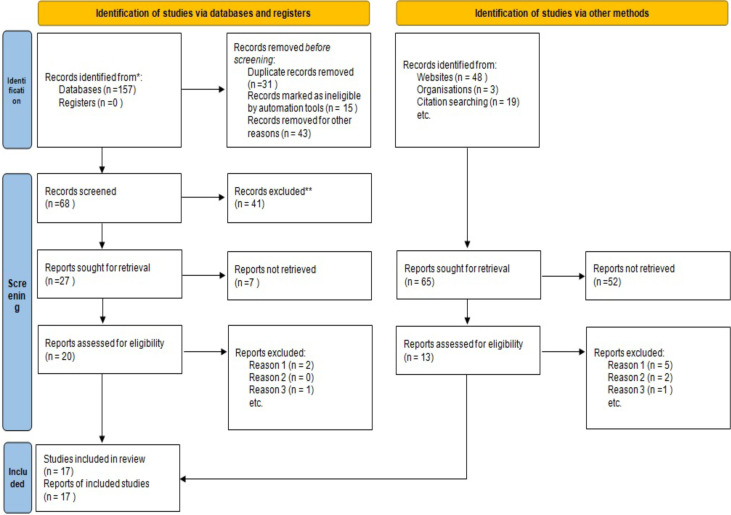
The flow-chart of the current systematic review and meta-analysis

In the initial search, we identified 157 documents by searching databases, as well as another 70 documents by searching other sources. The selection process is shown in Figure 1. Based on the inclusion/exclusion criteria, we finally enter 17 studies into the analysis in the present study (26–42). Table 1 shows the characteristics of included studies. The studies were conducted between 2004 and 2021. There were 8,767 study participants, out of which 8,237 were female and 530 were male.

**Table 1:** The characteristics of studies included

First author	Year	Q1	Q2	Q3	Q4	Q5	Q6	Q7	Q8	Q9	Q10
Kebriaei	2004	Yes	Yes	Yes	Unclear	Yes	Yes	Yes	Yes	Yes	Yes
Aghamolaei	2008	Yes	Yes	Yes	Yes	Unclear	Yes	Yes	Yes	Yes	Yes
Mohammadi	2009	Yes	Yes	Yes	Yes	Yes	Yes	Yes	Yes	Yes	Yes
Roohi	2011	Yes	Yes	Yes	Yes	Yes	Yes	Yes	Yes	Yes	Unclear
Gholami	2011	Yes	Yes	Yes	Unclear	Yes	Yes	Yes	Yes	Yes	Yes
Tarrahi	2012	Yes	Yes	Yes	Yes	No	Yes	Yes	Unclear	Yes	Yes
Ghanbari	2013	Yes	Yes	Yes	Yes	Yes	Yes	Yes	Yes	Yes	Yes
Safi	2014	Yes	Yes	Yes	Yes	Unclear	Yes	Yes	Yes	Yes	Yes
Vafaee-Najar	2014	Yes	Yes	Yes	Yes	Yes	Yes	Yes	Yes	Yes	Unclear
Tabatabaei	2015	Yes	Yes	Yes	Yes	Yes	Yes	Yes	Yes	Yes	Yes
Oliaee	2016	Yes	Yes	Yes	Unlcear	Yes	Yes	Yes	Yes	Yes	Yes
Kazemnezhad	2016	Yes	Yes	Yes	Yes	Yes	Yes	Yes	Yes	Yes	Unclear
Matin	2016	Yes	Yes	Yes	Yes	Yes	Yes	Yes	Yes	Yes	Yes
Gholipour	2019	Yes	Yes	Yes	Yes	Yes	Yes	Yes	Unclear	Yes	Yes
Gholipour	2019	Yes	Yes	Yes	Yes	Unclear	Yes	Yes	Yes	Yes	Yes
Sharifi	2021	Yes	Yes	Yes	Yes	Yes	Yes	Yes	Yes	Yes	Yes
Sharifirad	2021	Yes	Yes	Yes	Yes	Yes	Yes	Yes	Yes	Yes	Yes

Table 2 shows the quality assessment of included studies using CASP checklist.

**Table 2:** The quality assessment of studies included

First author	Year	City	No. Participants	No. Women	No. Men	Mean±SD
Kebriaei	2004	Kashan	300	300	0	29.16±7.07
Aghamolaei	2008	Bandarabbas	400	400	0	22.5
Mohammadi	2009	Zanjan	300	300	0	28.4
Roohi	2011	Gorgan	225	201	24	28±7.5
Gholami	2011	Neyshabour	400	400	0	32.54±11.99
Tarrahi	2012	Khoarramabad	650	621	29	28.5
Ghanbari	2013	Tehan	500	500	0	30.9±6.9
Safi	2014	Tehan	325	302	23	35.5
Vafaee-Najar	2014	Mashhad	435	387	48	30.30±10.37
Tabatabaei	2015	Zahedan	438	438	0	26.4 ± 5.9
Oliaee	2016	Isfahan	201	201	0	31.8 ± 7.3
Kazemnezhad	2016	Qom	409	409	0	27.70±5.98
Matin	2016	Kermanshah	400	13	387	NA
Gholipour	2019	Sanandaj	384	384	0	35
Gholipour	2019	Sanandaj	1920	1920	0	NA
Sharifi	2021	Mashhad	200	181	19	NA
Sharifirad	2021	Isfahan	1280	1280	0	27.53

**Perception**: Using a random model, we calculated the mean perception of the included studies (see Table 3). Also, total service quality for perception was 3.83 **[95% CI:** 3.50 to 4.15].

**Table 3:** Mean of perceptions in included studies using SERVQUAL in PHC in Iran

Dimensions	Mean with 95% CI	P-Value	I^2^	Publication bias
**Tangibles**	3.71 (3.41 to 4.01)	0.000	100%	0.34
**Reliability**	4 (3.64 to 4.35)	0.000	100%	0.29
**Responsiveness**	3.79 (3.43 to 4.15)	0.000	100%	0.11
**Assurance**	3.83 (3.57 to 4.09)	0.000	100%	0.53
**Empathy**	3.86 (3.44 to 4.29)	0.000	100%	0.19
**Total Service Quality**	**3.83 (3.50 to 4.15)**	**0.000**	**100%**	

**Expectation**: The mean expectation of the included studies are presented in Table 4. Tangibles and responsiveness had the highest and lowest mean. Also, total service quality for expectation was 4.36 [95% CI: 4.11 to 4.62].

**Table 4:** Mean of expectation in included studies using SERVQUAL in PHC in Iran

Dimensions	Mean with 95% CI	P-Value	I^2^	Publication bias
**Tangibles**	4.46 (4.28 to 4.65)	0.000	100%	0.29
**Reliability**	4.46 (4.25 to 4.67)	0.000	100%	0.17
**Responsiveness**	4.36 (4.05 to 4.66)	0.000	100%	0.31
**Assurance**	4.36 (4.05 to 4.67)	0.000	100%	0.72
**Empathy**	4.36 (4.15 to 4.36)	0.000	100%	0.13
**Total Service Quality**	**4.36 (4.11 to 4.62)**	**0.000**	**100%**	

**Quality gap**: The mean expectation of the included studies is as follows: Tangibles = -0.75, reliability = -0.46, responsiveness = -0.57, assurance = - 0.53, and empathy = -0.50. Also, the quality gap for total service quality was -0.53.

## Discussion

Improving health services is a vital issue for recipients and patients, service providers, and health policymakers. In this review study, we examined the quality of services provided in PHC, and the views of recipients of these services. Findings of this study show that there is a difference between the expectations and the perception of the services they have received in PHC centers in Iran (**-**0.53). Among the items of SERVQUAL instrument related to service recipients' perceptions, four items value less than 4; only one item had the value above 4; and in total, the service quality had a value of 3.83.

According to the results of this study, our finding is not consistent with that of Greece's study examining the quality of PHC services (43); this difference can be due to the structures of the health sector in various countries. The attention of policymakers and the importance they give to these services in the community can make providers improve their services over other centers. Meanwhile, an important point that we should consider is that all PHC centers in Iran are public, and the private sector is not active in this field (44). On the other hand, there is not much competition between the centers, and policymakers do not have a specific plan to evaluate the quality of these services. Private sector participation in the provision of primary health services seems to be one of the best options to increase the quality of services (44). Compared to the services provided in hospitals in Iran, the quality of PHC services is lower. One of the problems observed in the health sector in Iran is that people are not interested to receive services in PHC; instead, they prefer to visit hospitals to receive the same services. Despite the important role that PHC has in improving the level of health in Iran, policymakers have paid less attention to PHC in recent years, and have focused more on hospitals rather than PHC (45, 46).

In the present study, a negative gap was observed between expectation and perception in all dimensions of the SERVQUAL instrument, which was similar to the findings of other countries (47, 48). There is no study on this gap in PHC; however, some problems such as lack of manpower, insufficient salaries for service providers compared to hospital staff, insufficient attention to PHC functions, and lack of equipment and up-to-date electronic devices can be the reasons why providers are reluctant to increase the quality of services.

Our findings show that tangibles had the largest gap among other dimensions, and the lowest gap was for the empathy dimension. Regarding the tangibles dimension, due to the lack of financial resources in the health sector, many PHC buildings do not belong to the government; also, many PHC centers do not have adequate physical space, and due to the changes in physical space that takes place every year, it causes a lot of problems for service recipients (13). Therefore, the lack of equipment in PHC in Iran is tangible. Paying attention to the needs of service recipients is one of the points that affect empathy. The staff of PHC centers, due to their high scientific skills, try to meet the needs of service recipients in the shortest possible time (35, 49). Proper ethics is also one of the strengths of the staff in PHC (17). Service providers need to be well-aware of the needs of service recipients. It seems that the recipients of services in PHC centers do not have adequate information about all the services in these centers. One of the reasons could be the lack of proper information in this regard (50). On the other hand, the lack of appropriate information on the part of service recipients and asymmetric information lead to low quality services. By continuously evaluating the needs of service recipients and reducing asymmetric information, the quality of services can be improved (42).

Also, health policymakers in Iran should pay more attention to the physical space for PHC centers (36). In addition, health managers in Iran should equip all PHC centers with new and up-todate facilities. Fortunately, the willingness of health care providers to use technologies related to this sector is increasing (42).

We also observed gaps regarding the dimensions of assurance, empathy, reliability, and responsiveness that are related to interpersonal relationships in PHC centers between providers and clients. In this regard, Babakus and Mangold (51) believe that the gap in these dimensions indicates serious problems in service quality. The low values of these dimensions should be eliminated by continuously training service providers, increasing their rewards, and hiring new and specialized staff in order to improve the quality of services.

The present study had some limitations. First, the observed heterogeneity between the included studies was high, which could be due to differences in methodology and different contexts of the geographical locations of the studies. On the other hand, most of the study participants were women. Although PHC centers are for public use, women are the main recipients of their services. In this regard, it is claimed that women's expectations of health services are higher and different compared to men, which can also affect the findings of our study. Meanwhile, in many provinces in Iran, relevant studies have not been yet conducted in PHC centers.

Results of this study show that there is a significant gap between perceptions and expectations of service recipients in PHC centers in Iran. Despite the fact that policies related to PHC have been on the agenda of policymakers in Iran for many years, their activities and services have not been updated for long. Also, we believe that appropriate physical space should be provided for these centers. Meanwhile, hiring human resources with good salaries should be considered seriously. Also, more qualitative studies should be conducted on the needs of service providers and recipients in PHC in future.
